# Experimental and Numerical Investigation of Patch Repair for Composite Laminates Subjected to Low-Velocity Impact

**DOI:** 10.3390/polym17070942

**Published:** 2025-03-30

**Authors:** Xiaojun Wei, Mingxuan Huang, Chaocan Cai, Zhonghai Xu, Qingyu Peng

**Affiliations:** 1 National Key Laboratory of Science and Technology on Advanced Composites in Special Environments, Harbin Institute of Technology, Harbin 150080, China; weixiaojun017@163.com (X.W.); 22b918033@stu.hit.edu.cn (M.H.); pengqingyu@hit.edu.cn (Q.P.); 2 Suzhou Research Institute of HlT, Suzhou 215104, China

**Keywords:** composite material, patch repair, FEM

## Abstract

The widespread use of composite materials has led to an increased focus on restoring the mechanical properties of damaged composite structures to ensure system safety. This study combines compression experiments and finite element simulations to investigate the effectiveness of different patch sizes and repair methods, including single- and double-sided repairs, in restoring the structural strength of composite laminates with barely visible impact damage (BVID). The results demonstrate that low-velocity impact significantly affects the strength of the laminate, reducing it to 68.53% of its original strength, highlighting the necessity of patch repair. For composite specimens repaired using patching, an increase in the patch radius consistently enhances strength recovery, reaching up to 93.96% of the original strength. However, this also leads to an increase in weight, suggesting that the patch radius should be selected based on the specific requirements of the application. Furthermore, double-sided patching is preferable to single-sided patching. This approach improves the repair efficiency by 4.96%, primarily due to its ability to provide a more uniform stress distribution. Consequently, the risk of premature buckling and failure under compressive loading is significantly reduced, ensuring improved structural integrity and durability. The finite element simulation results presented in this study align well with the experimental findings, with a maximum error of no more than 10.68%. In conclusion, this work provides reliable guidance for the optimal patch repair of composite structures and lays a solid foundation for the practical application of patch repairs in engineering.

## 1. Introduction

The widespread adoption of composite materials in aerospace, automotive, and energy industries can be primarily attributed to their exceptional mechanical properties and lightweight characteristics, particularly those of carbon fiber-reinforced polymers (CFRPs) [1,2]. Due to their superior specific strength, specific stiffness, and fatigue resistance, CFRPs have increasingly replaced traditional materials such as steel and aluminum, becoming a critical component in aircraft structures [3]. For example, CFRPs constitute up to 53% of the structural components in the Airbus A350, including key elements such as the wings, fuselage, and tail [4,5].

During service, composite laminates are susceptible to low-velocity impacts (LVI), often resulting from debris impacts during takeoff, landing, and taxiing or from dropped tools during maintenance activities [6,7]. These impacts can cause barely visible impact damage (BVID), which may lead to delamination [8] and intralaminar damage [9]. Such damage significantly reduces composite structures’ compression-after-impact (CAI) strength, thereby compromising their structural integrity. To minimize maintenance costs, minor impact damage in composite structures is typically repaired rather than replaced [10]. Consequently, the development of effective repair techniques to restore the performance of composites after impact damage is critical for ensuring their continued serviceability [11,12].

Currently, the repair of composite structures is primarily carried out using either mechanical fasteners or adhesive patches [11]. Among these methods, mechanical fastening is widely used due to its advantages, such as ease of application and significant repair effectiveness [13]. However, this approach has some limitations: the addition of metal fasteners increases the overall weight of the structure and generates high-stress concentrations around the connection holes, leading to significantly lower durability compared to adhesive-bonded repairs [14]. Therefore, exploring strength restoration techniques for composite structures based on the patch repair method is of considerable research importance. Mudassir Ali et al. [15] proposed that patch repair could serve as a temporary solution for emergency repairs; however, if critical components are damaged, replacement or alternative repair methods should be considered. Caliskan U et al. [16] examined the strength of composite structures by investigating the impact of patch material and thickness on the repair process, confirming the feasibility of using patch repair to restore the performance of impact-damaged laminates. Several design parameters for patch repair, such as patch angle, have been extensively studied. Brighenti et al. [17] applied a biologically-inspired approach, a genetic algorithm, to optimize the shape of patches for repairing cracked plates. Their findings demonstrated that, for certain configurations, using an optimally shaped patch (rather than a simple square or rectangle) could significantly reduce the stress concentration factor.

On the other hand, bonded repairs minimize stress concentrations and offer high reinforcement efficiency, making them increasingly favored and gaining significant attention. Depending on the size of the damaged area and the requirements of the repaired structure, either patch repair or scarf repair techniques can be employed [18,19,20]. To optimize the parameters of patch repair, constructing a well-designed and effective finite element model is essential [21]. For instance, Psarras et al. [22] conducted tensile tests on composite specimens repaired using adhesive patches and developed and validated a numerical model based on the experimental results. This model provides a predictive tool for designing and implementing adhesive repairs in aerospace composite structures. Breitzman [23] investigated the effects of patch ply orientations, including nontraditional directions and the presence or absence of laminated layers, on the tensile performance of composite repairs. Through multidimensional optimization, the study identified ply orientations that minimized von Mises stresses in the adhesive. These optimized stacking sequences resulted in a significant reduction in stress levels and restored the repair strength to 85% and 90% for flush and single-thickness laminated repairs, respectively. In this paper, a finite element model of the post-patch composite was constructed, yielding a low error margin of no more than 10%.

In this study, compression damage tests were performed on post-impact composite laminates repaired using the patch repair method. The effectiveness of this repair technique on impact-damaged composites was evaluated by comparing the experimental results of intact, damaged, and repaired specimens under varying conditions. After repair, the strength recovery rate for single-side patching reached 81.61%, while double-side patching reached 88.13%, demonstrating the superior effectiveness of double-side patching in restoring structural strength. Furthermore, the impact of patch size on the repair performance was also investigated. The results indicated that increasing the patch radius enhances the repair effect. However, excessively large patches may lead to significant weight gain, highlighting the importance of carefully selecting the patch radius for practical applications.

## 2. Constitutive Model

Damage of composite laminates can be categorized into two types: intra-laminar damage and inter-laminar damage. Intra-laminar damage manifests as fiber breakage and matrix cracking, while inter-laminar damage primarily results from delamination and debonding. Therefore, the constitutive model employed in this study accounts for both intralaminar and interlaminar damage to effectively capture all potential damage behaviors in CFRP laminates under compressive behavior.

### 2.1. Basic Formula

For an ordinary composite laminate, the displacement and stress fields can be represented in the following form [24]:

(1) $$\begin{array}{c} U(x,y,z)=\left[\begin{array}{l} u(x,y,z)\\ v(x,y,z)\\ w(x,y,z) \end{array}\right]=\left[\begin{array}{l} u_{0}(x,y)+z\cdot \theta_{x}(x,y)\\ v_{0}(x,y)+z\cdot \theta_{y}(x,y)\\ w_{0}(x,y) \end{array}\right]\\ \varepsilon_{x}=\frac{\partial u_{0}}{\partial x}+z\cdot \frac{\partial \theta_{x}}{\partial x},\;\varepsilon_{y}=\frac{\partial v_{0}}{\partial y}+z\cdot \frac{\partial \theta_{y}}{\partial y} \end{array}$$

(2) $$\begin{array}{c} \gamma_{xy}=\frac{\partial u_{0}}{\partial y}+\frac{\partial v_{0}}{\partial x}+z\cdot (\frac{\partial \theta_{x}}{\partial y}+\frac{\partial \theta_{y}}{\partial x})\\ \gamma_{xz}=\frac{\partial w}{\partial x}+\theta_{x},\;\gamma_{yz}=\frac{\partial w}{\partial y}+\theta_{y} \end{array}$$

where, $u_{0},v_{0},w_{0}$ represents the displacement, and $\varepsilon_{x},\varepsilon_{y},\gamma_{xy}$ represents the strain, z is variable in z direction. The equations for calculating the stresses in the structure are then derived from the general form of the constitutive equations [25]:

(3) $$\left\{\begin{array}{c} N_{x}\\ N_{y}\\ N_{xy} \end{array}\right\}=\left[\begin{array}{ccc} A_{11} & A_{12} & A_{16}\\ A_{12} & A_{22} & A_{26}\\ A_{16} & A_{26} & A_{66} \end{array}\right]\left\{\begin{array}{c} \varepsilon_{x}^{0}\\ \varepsilon_{y}^{0}\\ \gamma_{xy}^{0} \end{array}\right\}+\left[\begin{array}{ccc} B_{11} & B_{12} & B_{16}\\ B_{12} & B_{22} & B_{26}\\ B_{16} & B_{26} & B_{66} \end{array}\right]\left\{\begin{array}{c} k_{x}^{0}\\ k_{y}^{0}\\ k_{xy}^{0} \end{array}\right\}$$

(4) $$\left\{\begin{array}{c} M_{x}\\ M_{y}\\ M_{xy} \end{array}\right\}=\left[\begin{array}{ccc} B_{11} & B_{12} & B_{16}\\ B_{12} & B_{22} & B_{26}\\ B_{16} & B_{26} & B_{66} \end{array}\right]\left\{\begin{array}{c} \varepsilon_{x}^{0}\\ \varepsilon_{y}^{0}\\ \gamma_{xy}^{0} \end{array}\right\}+\left[\begin{array}{ccc} D_{11} & D_{12} & D_{16}\\ D_{12} & D_{22} & D_{26}\\ D_{16} & D_{26} & D_{66} \end{array}\right]\left\{\begin{array}{c} k_{x}^{0}\\ k_{y}^{0}\\ k_{xy}^{0} \end{array}\right\}$$

where, $k_{x}^{0},\;k_{y}^{0}$ and $k_{xy}^{0}$ represents curvature, $N_{x},\;N_{y},\;N_{xy}$ and $M_{x},\;M_{y},\;M_{xy}$ represents the internal force and moment of the laminate, respectively, $A_{ij},\;B_{ij},\;D_{ij}$ represent in-plane stiffness, coupling stiffness, and bending stiffness, respectively.

### 2.2. Intra-Laminar Damage

The stiffness degradation of the impact-damaged region of the composite laminate is analyzed using the Camanho material degradation model. Hashin damage criterion [26] is utilized to predict the onset of intralaminar damage and the progressive failure of repaired composite laminates. The Continuum Damage Model (CDM) is employed to calculate the residual strength of damaged laminates. Once intralaminar damage occurs, CDM is used to model the corresponding damage evolution.

In accordance with the various damage modes, the corresponding degradation factors are selected. By employing matrix cracking and delamination degradation parameters, the material’s elastic constants are reduced in order to simulate the damage caused by low-velocity impact. The specific degradation modes of the Camanho material degradation model are as follows [27]:

Matrix tensile fracture:

(5) $$E_{2}^{d}=0.24E_{2}^{0},\;G_{12}^{d}=0.2G_{12}^{0},\;G_{23}^{d}=0.2G_{23}^{0}$$

Matrix compression fracture:

(6) $$E_{2}^{d}=0.4E_{2}^{0},\;G_{12}^{d}=0.4G_{12}^{0},\;G_{23}^{d}=0.4G_{23}^{0}$$

Fiber tensile fracture:

(7) $$E_{1}^{d}=0.07E_{1}^{0}\;$$

Fiber compressive fracture:

(8) $$E_{1}^{d}=0.14E_{1}^{0}\;$$

Delamination:

(9) $$E_{3}^{d}=G_{13}^{d}=G_{23}^{d}=\nu_{13}^{d}=\nu_{23}^{d}=0$$

where *E*_i_ (i = 1, 2, 3) represents the Young’s modulus along the principal directions of the material, *G*_ij_ (i,j = 1, 2, 3) represents the shear modulus on the i-j plane, and *ν*_ij_ (i,j = 1, 2, 3) represents the Poisson’s ratio for the transverse deformation caused by stress in the principal direction iii on the principal direction j. The superscripts d and 0 denote the material constants after damage and the initial material constants, respectively.

Based on the Hashin damage criterion, the onset of damage to fibers and the matrix under tension and compression can be accurately predicted for each mode. Equations (10)–(13) account for four distinct failure criteria:

Fiber tension failure $\left(\sigma_{11}\geq 0\right)$:

(10) $$F_{ft}={\left(\frac{\sigma_{11}}{X_{T}}\right)}^{2}+\alpha {\left(\frac{\sigma_{12}}{S_{L}}\right)}^{2}=1$$

Fiber compression failure $\left(\sigma_{11}<0\right)$:

(11) $$F_{\mathrm{fc}}={\left(\frac{\sigma_{11}}{X_{C}}\right)}^{2}=1$$

Matrix tension failure $\left(\sigma_{22}+\sigma_{33}\geq 0\right)$:

(12) $$F_{\mathrm{mt}}={\left(\frac{\sigma_{22}}{Y_{T}}\right)}^{2}+{\left(\frac{\sigma_{12}}{S_{L}}\right)}^{2}=1$$

Matrix compression failure $\left(\sigma_{22}+\sigma_{33}<0\right)$:

(13) $$F_{\mathrm{mc}}={\left(\frac{\sigma_{22}}{2S_{T}}\right)}^{2}+\left[{\left(\frac{Y_{C}}{2S_{T}}\right)}^{2}-1\right]\frac{\sigma_{22}}{Y_{C}}+{\left(\frac{\sigma_{12}}{S_{L}}\right)}^{2}=1$$

where σ_11_, σ_22_ and σ_12_ represent the applied stresses. X_T_, X_C_, Y_T_, and Y_C_ represent the tensile and compressive strengths in fiber direction and matrix direction, respectively, while S_L_ and S_T_ represent the longitudinal and transverse shear strengths. The α (0.0 < α < 1.0) coefficient considers the contribution of the shear stress σ_12_ to the fiber tensile initiation criterion.

**Figure 1:**
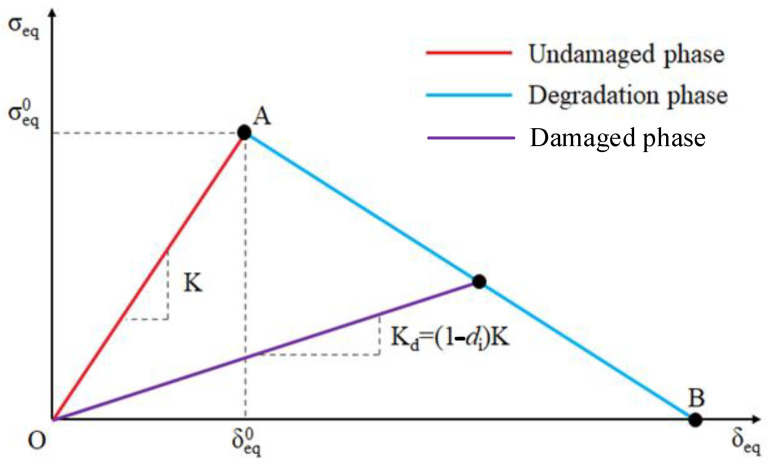
Linear degradation model.

Figure 1 illustrates the damage evolution under different failure modes. The undamaged phase is characterized by the initial material stiffness K. At point A, the Hashin criterion is satisfied. The segment AB represents the damage evolution phase up to point B, which corresponds to complete damage. Along segment AB, the stiffness decreases as a function of the degradation coefficient *d*, as described by Equation (14)

(14) $$d=\frac{\delta_{eq}^{f}(\delta_{eq}-\delta_{eq}^{0})}{\delta_{eq}(\delta_{eq}^{f}-\delta_{eq}^{0})}$$

where $\delta_{eq}^{0}$ is the initial equivalent displacement at which the initiation criterion for that mode was met, and $\delta_{eq}^{f}$ is the displacement at which the material is completely damaged in this failure mode.

### 2.3. Inter-Laminar Damage

The Cohesive Zone Model (CZM) is used to describe delamination between different layers of composite laminates as well as debonding between the damaged laminate and the repair patch [24]. A linear elastic traction-separation behavior is the initial constitutive behavior followed by the initiation and evolution of damage. The elastic behavior can be written as:

(15) $$T=\left\{\begin{array}{c} T_{n}\\ T_{s}\\ T_{t} \end{array}\right\}=\left[\begin{array}{ccc} E_{nn} & & \\ & E_{ss} & \\ & & E_{tt} \end{array}\right]\left\{\begin{array}{c} \varepsilon_{n}\\ \varepsilon_{s}\\ \varepsilon_{t} \end{array}\right\}$$

where the nominal traction stress vector *T* consists of three components: *T_n_*, *T_s_*, and *T_t_*, which represent the nominal tractions in the normal and two local shear directions, respectively. Similarly, *ε_n_*, *ε_s_*, and *ε_t_* are the corresponding nominal strains.

Inter-laminar damage is governed by a quadratic separation law. According to this law, delamination and debonding occur when the quadratic interaction function reaches a value of 1, as expressed by Equation (16) [8]:

(16) $${\left(\frac{\langle t_{n}\rangle }{T_{n}}\right)}^{2}+{\left(\frac{t_{s}}{S_{s}}\right)}^{2}+{\left(\frac{t_{t}}{S_{t}}\right)}^{2}=1$$

where, *t_n_*, *t_s_* and *t_t_* are the normal and shear stress; *T_n_*, *S_s_* and *S_t_* are the interface normal and shear strength, respectively.

**Figure 2:**
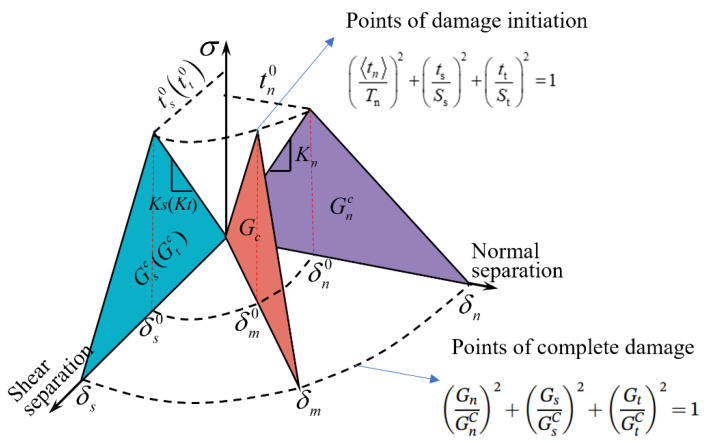
Bilinear traction separation law in mixed mode.

The damage evolution of cohesive elements follows an energy-based mixed-mode power law, as shown in Equation (17). This is illustrated schematically in Figure 2. The two vertical coordinate planes represent the response of cohesive elements under pure normal stress and pure shear stress states. $G_{n}^{C}$, $G_{s}^{C}$ and $G_{t}^{C}$ represent the fracture toughnesses for the three pure damage modes I, II, and III, respectively.

(17) $${\left(\frac{G_{n}}{G_{n}^{C}}\right)}^{2}+{\left(\frac{G_{s}}{G_{s}^{C}}\right)}^{2}+{\left(\frac{G_{t}}{G_{t}^{C}}\right)}^{2}=1$$

## 3. Experimental Preparation

### 3.1. Material and Specimen Preparation

**Figure 3:**
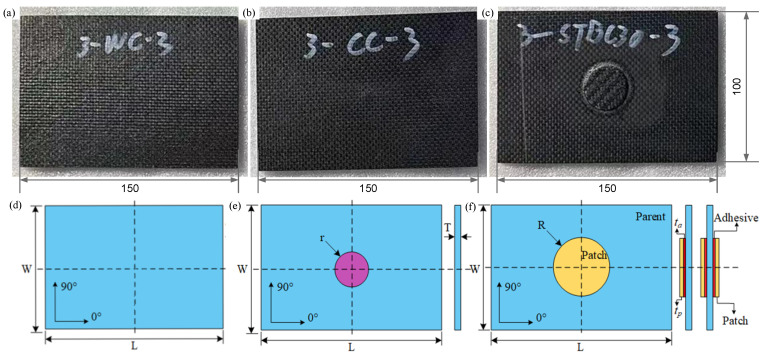
Geometry information of pristine (**a**,**d**), damaged (**b**,**e**), and patch-repaired CFRP laminates (**c**,**f**).

This paper analyzes three types of CFRP composite laminates: pristine laminate (PL), damaged laminate (DL), and patch-repaired laminate (PRL). PRL includes two types of laminate: single-sided patch repair laminate (SSPRL) and double-sided repair laminate (DSPRL). These laminates are fabricated using FP800/IS1801 carbon fiber/epoxy prepreg, with each ply having a thickness of *t*_p_ = 0.2 mm, and the fabricated specimen is shown in Figure 3a–c. The length (L), width (W), and thickness (T) of the composite laminate are 150 mm, 100 mm, and 3.2 mm, respectively, and the stacking sequence is [90]_16_, with fiber orientations defined as shown in Figure 3. The circular area (purple) with the radius r = 10 mm in Figure 3e is the local damage caused by the low-speed impact of the drop hammer, which is regarded as BVID. PRL consists of three parts: the parent laminate(blue), the patch (yellow), and the adhesive (red), as demonstrated in Figure 3f.

To compare the actual performance restoration of two commonly used repair strategies in engineering, single-side repair (SSPR) and double-side repair (DSPR), this study employs an adhesive-bonded repair technique for laminate restoration. The repair procedure consists of the following steps:

1. Surface preparation: The surface of the laminate containing impact damage was sanded, and the target area was rinsed with acetone to enhance adhesive bonding performance.
2. Patch application: A specified number of layers of 0° unidirectional prepregs were cut into circular patches of appropriate dimensions. The patches were then cured using the vacuum bagging method following standard autoclaving procedures. To optimize the bonding performance, resin bonding was employed to affix the patch to the laminate.
3. Final processing: Upon completion of the curing process, the vacuum bag was removed, and any excess adhesive was eliminated to prepare the specimen for experimentation.

In this study, the patch radius is set as R = 20 mm, and the adhesive layer thickness is defined as *t*_a_ = 0.4 mm in the finite element model. The schematic illustration of the repaired laminates with the patch is shown in Figure 3b. The mechanical properties of the composite laminates and adhesives were supplied by Weihai Expand Fiber Co., Ltd. (Weihai, China) and are presented in detail in Table 1.

### 3.2. Low Velocity Impact Test

**Figure 4:**
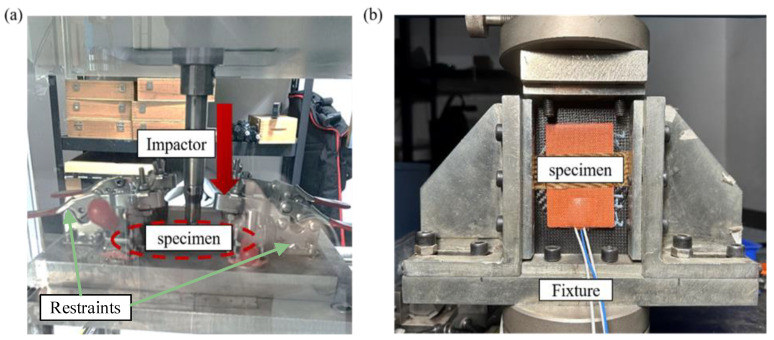
Experimental setup of LVI test (**a**) and Compression test (**b**).

According to the ASTM D7136/D7136-07 [28] standard, the LV1 test of PL was conducted using an INSTRON CEAST 9350 drop-weight experimental platform. It is composed of an impactor, a force sensor, a data acquisition system, and an anti-rebound mechanism, as shown in Figure 4a. The hemispherical impactor has a diameter of 16 mm and a mass of 5.482 kg. The control system set the initial velocities to 1.91 m/s and 2.339 m/s, corresponding to impact energies of 10 J and 15 J, respectively. The anti-secondary impact device prevented the impactor from causing secondary impacts on the specimen.

The impact energy history curve *E*(t) absorbed by the specimen was calculated using the force-time curve *F*(t), as shown by the following Equation (18)

(18) $$E_{a}(t)=E_{k0}-E_{k}(t)=E_{k0}-\frac{1}{2}m{\left[\nu_{0}-\frac{1}{m}\int_{0}^{t}F(t)dt\right]}^{2}$$

where, *m* and *E_k_*(*t*) represent the mass of the impactor and its instantaneous kinetic energy, respectively. The final absorbed energy, *E_a_*, is determined when the energy history curve shows a convergent value.

### 3.3. Compression Test

To evaluate the residual strength of the CFRP laminates, compression tests were conducted on PL, DL, and PRL specimens in accordance with ASTM D3410/D3410M [29]. To ensure the representativeness of the experimental data, each test group comprised three specimens. The compression test was conducted using a WDW-E200D computer-controlled electronic universal testing machine, with the experimental setup illustrated in Figure 4b. The testing system comprised an upper clamping platform, a lower support platform, and a slide rail. Equipped with high-precision built-in sensors, the machine accurately captured real-time variations in load and displacement during the compression test while automating the processing and output of experimental data. Additionally, photographs of the specimens before and after testing were taken using a Nikon D5300 camera to document the deformation and failure characteristics.

## 4. Finite Element Model

**Figure 5:**
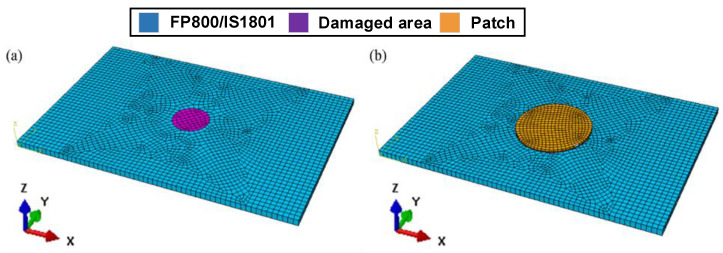
Finite Element Model of (**a**) DL and (**b**) PRL.

The 3D finite element model of compression was created in Abaqus based on the geometry shown in Figure 5. Based on the results of the impact experiment, the radius of the damaged region (purple) was determined to be 10 mm. Stiffness reduction was applied to this region following the progressive damage accumulation analysis method for composite laminates proposed by Wen [6].

**Figure 6:**
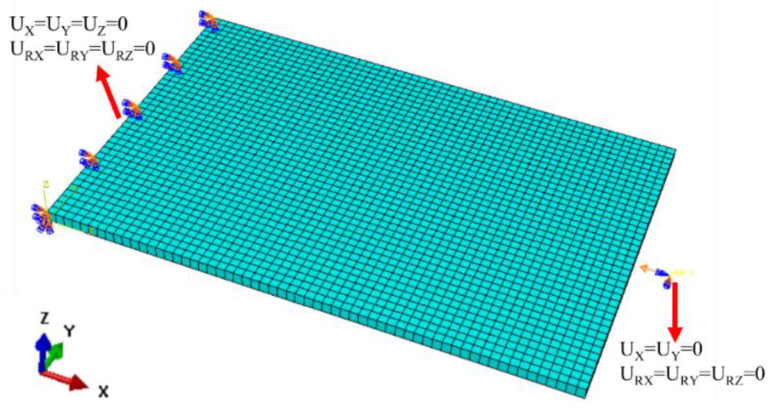
Finite Element Model and boundary conditions of PL.

Figure 6 presents the meshed finite element model employed for the compression simulation, which incorporates a repaired specimen featuring a circular patch with a thickness of 1 mm. Eight-node 3D solid, reduced integration elements (element type in Abaqus: C3D8R) were used to mesh the specimen. Moreover, cohesive elements (element type: COH3D8) were used to describe the adhesive interface between the parent plate and the patch. For the standard compression test fixture used in this study, a compressive load should be applied to the upper side of the simulated model, while the lower side should be fully constrained. The mesh and boundary conditions of PL are illustrated in Figure 6.

## 5. Result and Discussion

### 5.1. Determination of Impact Energy

In order to determine the appropriate impact energy for inducing BVID, low-velocity impact tests with varying energy levels were conducted on pristine composite laminates. In engineering practice, impact damage that results in a surface dent without full penetration is the most prevalent form of structural damage. For the laminate thickness considered in this study, impact energies of 10 J and 15 J are most likely to replicate similar damage conditions [1]. To mitigate experimental variability, each impact energy level was tested using two specimens to ensure the repeatability and reliability of the results. Specimens 1 and 2 were subjected to an impact energy of 10 J, while specimens 3 and 4 were subjected to 15 J.

**Figure 7:**
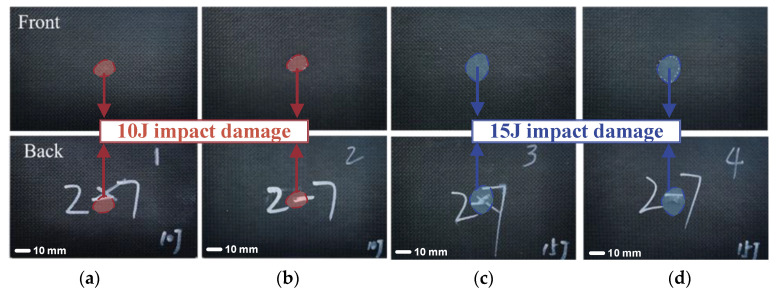
Composite laminates after impact ((**a**–**d**) correspond to specimens 1, 2, 3, and 4, respectively).

Figure 7 presents images of the composite laminates after impact at different energy levels. By comparing the photos of the front and back sides of the composite laminates after impact, it can be observed that the laminates subjected to 15 J impact sustained more severe damage, with perforation occurring on the backside (see Figure 7a,b). In contrast, the panels impacted with 10 J exhibited minimal damage, with no perforation observed (see Figure 7c,d).

**Figure 8:**
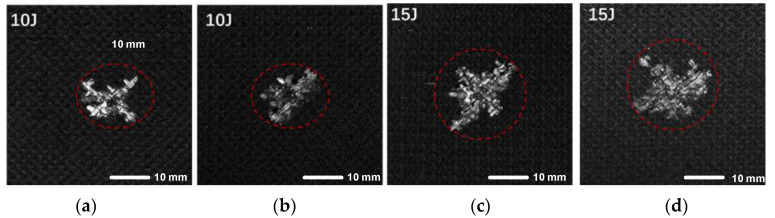
C-sweep results of composite laminates after impact ((**a**–**d**) correspond to specimens 1, 2, 3, and 4, respectively).

The specific dimensions of the damage were determined using C-scan imaging, as shown in Figure 8, with the damaged areas marked by red circles. The irregular shape of the damage indicates that the composite laminates experienced non-uniform failure during the impact, with the damage points distributed radially. Under the 15 J impact energy, the damage to the composite structure was significantly more severe than that under the 10 J impact energy. Specifically, the maximum damage radius centered around the impact point was 13.32 mm under the 15 J impact, while the maximum damage radius was 10 mm under the 10 J impact. To ensure that the damage morphology of the specimens selected for repair aligns with general engineering practice, an impact energy of 10 J was determined to be appropriate for inducing barely visible impact damage (BVID) in the composite structure. This selection was based on a comprehensive assessment of the impact damage characteristics of the composite material and the repair conditions.

### 5.2. Comparative Analysis of Limit Load and Damage Mode

#### 5.2.1. Pristine Composite Laminate

**Figure 9:**
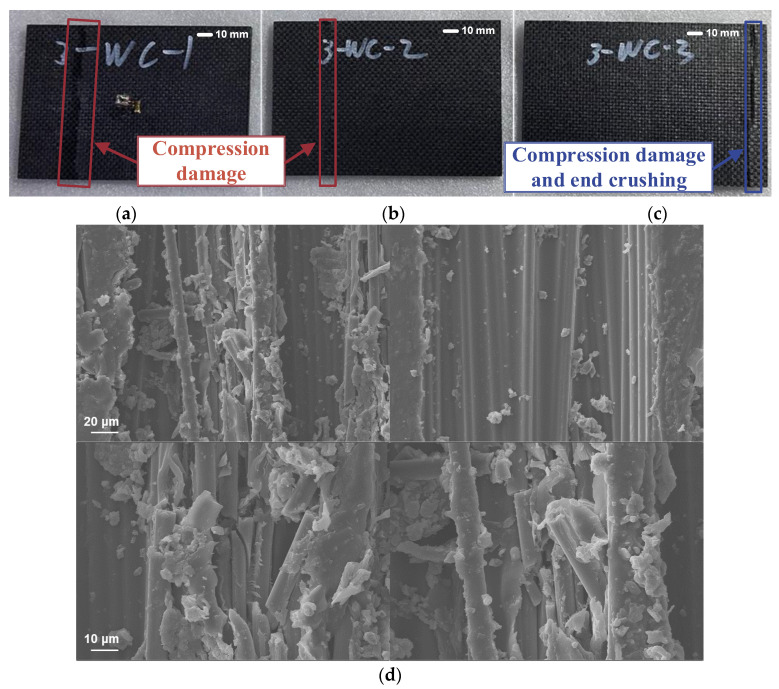
Pristine composite laminates after compression test ((**a**–**c**) correspond to specimens 1, 2, and 3, respectively, (**d**) is the SEM result).

The pristine laminate was first subjected to a compression failure test to measure its ultimate strength. The image of damage after the test is shown in Figure 9d. During the tests, it was observed that although the location of the damage was different for each specimen, the mode of failure was similar. For specimen WC-3, the right side experienced end crushing, but the crushing occurred away from the boundary, which can be considered a reasonable form of damage. In the other specimens, the damage was mainly to the left of the central area. Therefore, the failure modes in all three tests were considered reasonable. During the test, the left central region of the specimen was first crushed, followed by delamination and fiber break. These fractures propagated through the full thickness of the specimen and then rapidly spread to both sides, resulting in the complete failure of the specimen. Figure 9d presents the scanning electron microscope (SEM) analysis of WC-1, revealing that the primary damage mechanism is fiber debonding induced by compressive loading. Additionally, some fibers exhibit brittle fracture due to ply flexure; however, the deeper fibers remain largely intact. These observations suggest that patch repair can effectively restore the surface strength of the composite structure, thereby enhancing its overall structural safety and integrity.

FEM results showed that the ultimate strength of the specimen was 93.45 kN. The average strength measured in the test was 85.92 kN, with an error of 8.7% compared to the simulated results, indicating a strong agreement between the experimental and simulated results. For pristine specimens, the experimental and simulation results are shown in Table 2.

#### 5.2.2. Damaged Composite Laminate

**Figure 10:**
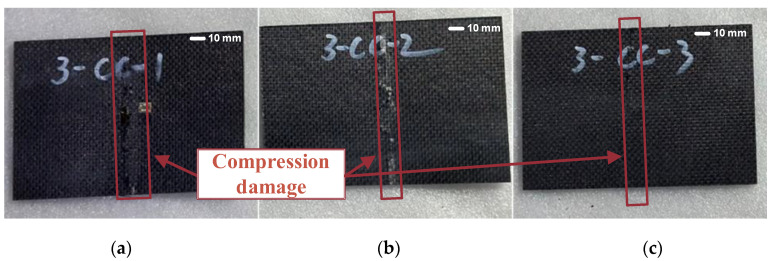
Damaged composite laminates after compression test ((**a**–**c**) correspond to specimens 1, 2, and 3, respectively).

Compression tests were then carried out on the damaged composite laminates, and the failure modes are shown in Figure 10. Samples CC-1 and CC-2 exhibited severe compression damage at the center of the laminate, while sample CC-3 showed only minor compression damage at the center. In this study, the term compression damage is used as a broad descriptor encompassing fiber debonding, fiber breakage, and interlaminar delamination induced by compressive loading. During the experiment, it was observed that the damage in all impacted laminates began at the damaged area and gradually propagated toward the laminate boundaries, eventually resulting in complete laminate failure.

**Figure 11:**
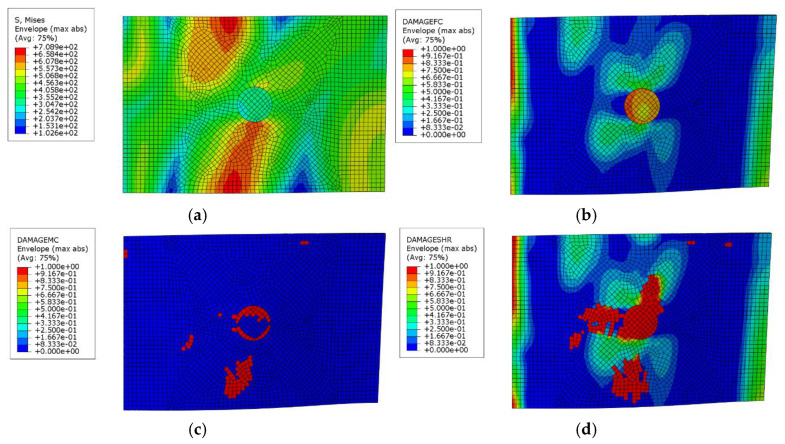
Simulation results for damaged composite laminates after compression test ((**a**) Stress distribution map, (**b**) Fiber damage distribution map, (**c**) Matrix damage distribution map, and (**d**) Shear damage distribution map).

According to the simulation results shown in Figure 11, the damage in the composite laminate during compression primarily occurs near the impact site and propagates outward from the edges of the impact damage. Fiber compression damage and shear damage are particularly severe, ultimately leading to the failure of the entire laminate. The stress distribution map further reveals that the central region of the laminate experiences higher stress, indicating that the laminate is more susceptible to damage in areas with higher stress concentrations, represented by the more intense (red) regions in the image during compression.

In Table 3, the finite element simulation results showed a strength of 50.20 kN, while the average measured strength in the experiment was 56.2 kN with an error of 10.68%, which is less than 15%, indicating that the simulation results are reasonable and in agreement with the experimental data. The strength of the damaged composite laminate is only 68.53% of that of the pristine composite laminate, indicating that BVID significantly reduces the mechanical properties of composite laminates. Therefore, the timely repair of damaged composite structural components is critical.

#### 5.2.3. Repaired Composite Laminate

**Figure 12:**
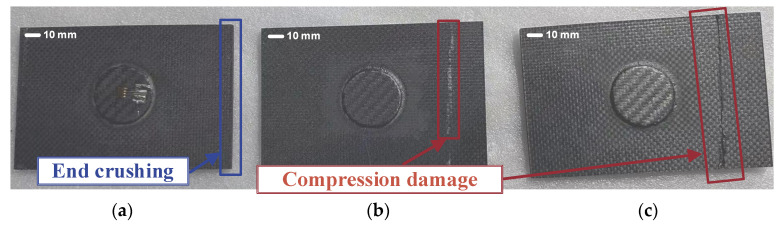
Single-sided patch repair composite laminates after compression test ((**a**–**c**) correspond to specimens 1, 2, and 3, respectively).

After the patch repair, compression tests were carried out on the composite laminates. The failure modes of the composite laminates repaired with single-sided patches are shown in Figure 12. Specimen 1 showed crushing damage at the edge, while specimens 2 and 3 both showed compression failure near the center-left area. The final damage locations for all specimens bypassed the patched areas, indicating that the repaired patch area has significantly higher strength.

**Figure 13:**
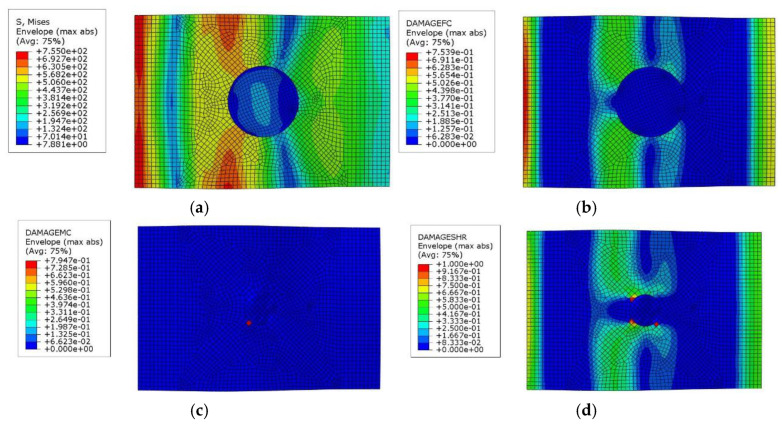
Simulation results for single-sided patch repair composite laminates after compression test ((**a**) Stress distribution map, (**b**) Fiber damage distribution map, (**c**) Matrix damage distribution map, and (**d**) Shear damage distribution map).

The simulation results are in good agreement with the experimentally observed damage patterns, as shown in Figure 13. Furthermore, fiber compression damage and shear damage are the most severe. Notably, the fiber damage extends directly through the middle of the laminate, which aligns with the experimental findings.

In Table 4, the finite element simulation results showed a strength of 71.47 kN, while the average measured strength in the experiment was 70.12 kN with an error of 1.93%, indicating a high degree of agreement between the simulated and experimental results. The strength of the damaged laminates is only 68.53% of that of the pristine laminates, while the strength of the laminates repaired with a single-sided patch is 81.61% of that of the pristine laminates, demonstrating high repair efficiency. This indicates that single-sided patching can restore the mechanical properties of the composite structure to some extent.

**Figure 14:**
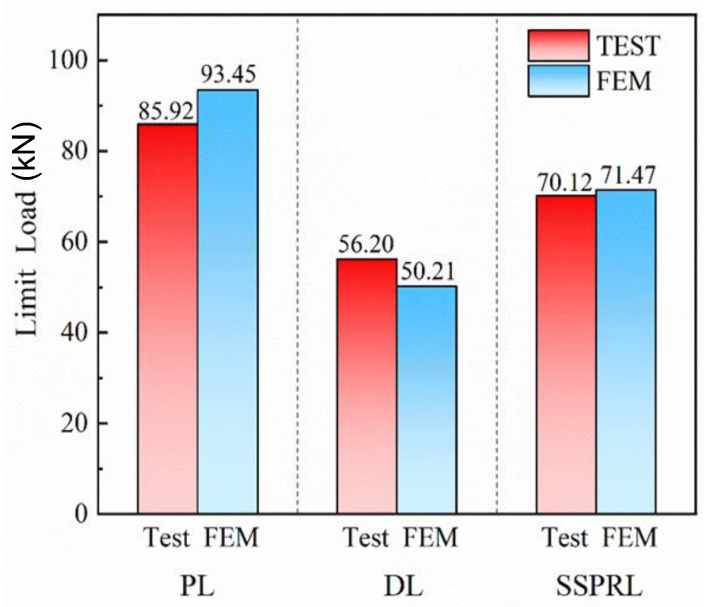
Comparison of experimental and simulation results.

Figure 14 presents a comparison of all experimental and simulation results in this study. It is evident that the finite element simulation results closely align with the experimental data, with a maximum error of no more than 10.68%, regardless of the repair method. Additionally, the results show that impact damage significantly reduces the mechanical properties of the laminate, while patch repair improves the structural strength by approximately 13.08%. This highlights the effectiveness of patch repair in enhancing the safety of the structure.

### 5.3. Effect of Patch Parameters on the Repair Performance

#### 5.3.1. Effect of Repair Methods

In this study, the effect of single-sided and double-sided repairs on the repair performance is investigated by changing the repair method. The patch radius is R = 20 mm. To exclude the interference of other parameters, the patch lay-up sequence for the double-sided repair is [0]_4_, and the patch lay-up sequence for the single-sided repair is [0]_8_, ensuring that the total thickness of the patch is the same. The strength analysis of the single-sided repaired laminate has been discussed in Section 5.2.3 and will not be repeated here.

**Figure 15:**
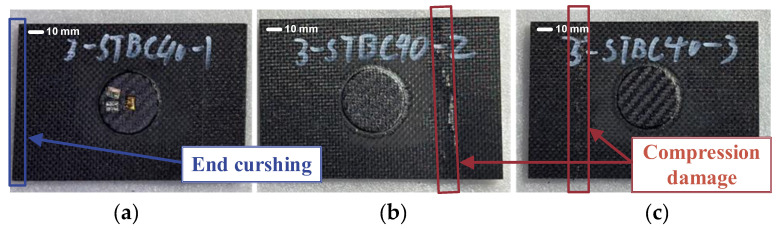
Double-sided patch repair composite laminates after compression test ((**a**–**c**) correspond to specimens 1, 2, and 3, respectively).

The failure mode of the double-sided patch-repaired laminate is shown in Figure 15. The failures of specimens 1 and 3 are relatively inconspicuous, while specimen 2 exhibits a more typical failure mode, with noticeable compressive damage on the right side of the laminate. This is a reasonable form of damage. The final failure locations of all specimens bypassed the repair area, which is consistent with the damage pattern observed in single-sided repairs. Table 5 shows that the strength obtained from the finite element simulation is 79.78 kN, whereas the average strength measured experimentally is 75.72 kN, resulting in a 5.36% error. This indicates a high degree of agreement between the simulation and experimental results, confirming the accuracy of the model.

**Figure 16:**
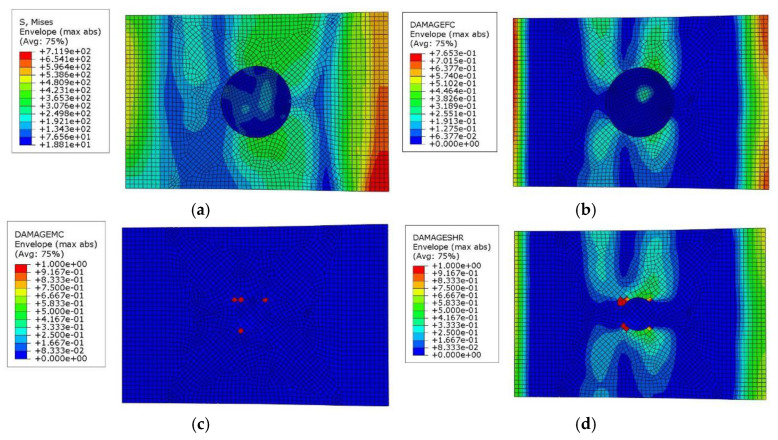
Simulation results for double-sided patch repair composite laminates after compression test ((**a**) Stress distribution map, (**b**) Fiber damage distribution map, (**c**) Matrix damage distribution map, and (**d**) Shear damage distribution map).

The simulation results, shown in Figure 16, reveal that fiber compression damage and shear damage are the most severe. Notably, the fiber damage directly penetrates the middle of the laminate, which is consistent with the experimental results. The fiber shear damage distribution map further shows significant damage occurring at the boundary of the impact zone, reflecting the actual experimental observations.

**Figure 17:**
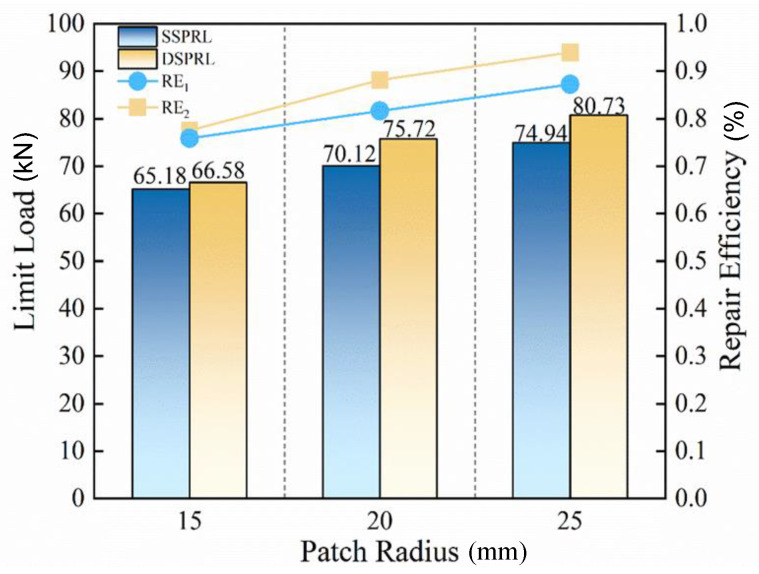
Comparison of test results.

To provide a more comprehensive characterization of the effect of different repair schemes on the structural strength restoration of damaged composites, experiments on single- and double-sided patch repairs at three patch radii were conducted, as shown in Figure 17. The results indicate that, regardless of the patch radius, the repair effectiveness of double-sided patching is superior to that of single-sided patching, with an average improvement of 4.96%. Therefore, in practical repair applications, double-sided patching should be preferred to restore the mechanical properties of composite structures.

#### 5.3.2. Effect of Patch Size

The patch size was primarily determined by the radius. The effect of patch size on repair performance was investigated by varying the patch radius. To eliminate the influence of other parameters on repair performance, circular patches with the same number of layers were fabricated, and the lay-up angle was fixed at 0 degrees. Compression tests were performed on specimens with three different patch radii, corresponding to 1.5, 2, and 3 times the damage radius. Given that the damage radius is r = 10 mm, the patch radii are 15 mm, 20 mm, and 25 mm, respectively.

The experimental results are shown in Figure 17. For a patch radius of R = 15 mm, the ultimate strength of SSPRL is 65.15 kN, with a repair efficiency of 75.86%. The ultimate strength of DSPRL is 66.58 kN, with a repair efficiency of 77.49%. For a patch radius of R = 20 mm, the ultimate strength of SSPRL is 70.12 kN, with a repair efficiency of 81.61%. The ultimate strength of DSPRL is 75.72 kN, with a repair efficiency of 88.13%. For a patch radius of r = 25 mm, the ultimate strength of SSPRL is 74.94 kN, with a repair efficiency of 87.22%. The ultimate strength of DSPRL is 80.73 kN, with a repair efficiency of 93.96%. Results indicate that, regardless of whether the repair is single-sided or double-sided, the repair efficiency increases with the patch radius.

However, it should be noted that the increase in patch radius also leads to a significant increase in patch weight, which may be detrimental to the reduction of aircraft weight. Therefore, in practical applications, the patch size should be chosen appropriately based on specific requirements.

## 6. Conclusions

In this study, different patch sizes and both single-sided and double-sided patch repair methods were applied to repair composite laminates with barely visible impact damage (BVID). Compression tests and numerical simulations were conducted on the repaired laminates to investigate the effects of patch size and repair method on the repair performance. The main conclusions are as follows:

1. Barely visible damage, such as low-velocity impact (LVI) damage, has a profound effect on laminate strength, with the post-impact strength being only 68.53% of the original laminate strength. This highlights the substantial reduction in performance that occurs after impact damage.
2. For the same total patch thickness, double-sided repairs are generally more effective than single-sided repairs, resulting in an average strength recovery increase of 4.96%. This improvement is likely due to the more uniform stress distribution achieved with double-sided repairs, which helps prevent premature buckling and failure during compression.
3. The repair performance improves significantly with an increase in the repair radius. Regardless of the repair method, the effectiveness of the repair gradually increases with the patch radius, reaching up to 93.96%. However, the patch radius should be carefully selected to balance the potential weight gain of the structure.

## Figures and Tables

**Table 1:** Mechanical properties of materials.

Properties	FP800/IS1801	Properties	Adhesive
*ρ* (g/cm^3^)	1.6	*ρ* (g/cm^3^)	1.6
*E*_1_ (GPa)	62	*E*_nn_ (MPa)	3410
*E*_2_ (GPa)	62	*E*_ss_, *E*_ss_ (MPa),	1250
*ν*_12_	0.08	$T_{n}^{C}\;$ (MPa)	5.71
*G*_12_	3.787	$T_{s}^{C}\;\;\;T_{t}^{C\;}$ (MPa)	39.91
*G*_23_, *G*_13_ (MPa)	2.931	$G_{n}^{C\;}$ (MPa)	0.267
*X*_T_ (MPa)	820	$G_{s}^{C},G_{t}^{C\;}$ (MPa)	0.807
*X*_C_ (MPa)	565	-	-
*Y*_T_ (MPa)	820	-	-
*Y*_C_ (MPa)	565	-	-
*S*_12_, *S*_23_ (MPa)	62	-	-

**Table 2:** Bearing capacity of pristine composite laminates.

Specimen	Test Results/kN		FEM Results/kN	Error
	Limit Load	Average		
WC-1	94.51	85.92	93.45	8.7%
WC-2	80.02			
WC-3	83.22			

**Table 3:** Bearing capacity of damaged composite laminates.

Specimen	Test Results/kN		FEM Results/kN	Error
	Limit Load	Average		
CC-1	49.46	56.20	50.20	10.68%
CC-2	51.04			
CC-3	68.10			

**Table 4:** Bearing capacity of single-sided patch repair composite laminates.

Specimen	Test Results/kN		FEM Results/kN	Error
	Limit Load	Average		
DTBC-1	69.01	70.12	71.47	1.93%
DTBC-2	71.35			
DTBC-3	70.00			

**Table 5:** Bearing capacity of double-sided patch repair composite laminates.

Specimen	Test Results/kN		FEM Results/kN	Error
	Limit Load	Average		
STBC-1	70.50	75.72	79.78	5.36%
STBC-2	83.27			
STBC-3	73.40			

## Data Availability

The original contributions presented in this study are included in the article. Further inquiries can be directed to the corresponding authors.

## References

[1] Hu C., Xu Z., Chen D., Huang M., Cai C., Qiu J., He X. (2024). A novel integrated modeling strategy for predicting damage mechanisms and energy dissipation of composite stiffened structures under low-velocity impact and compression. Aerosp. Sci. Technol..

[2] Huang M., Xu Z., Hu C., Qiu J., Yin W., Wang R., He X. (2024). A method for detection of delamination depth position within composite laminates based on 2D continuous wavelet transform and CNN. Struct. Health Monit..

[3] Albat A.M., Romilly D.P., Raizenne M.D. (2000). Thermal residual stresses in bonded composite repairs on cracked metal structures. Mater. Technol..

[4] Umamaheswar T., Singh R. (1999). Modelling of a patch repair to a thin cracked sheet. Eng. Fract. Mech..

[5] Clark R.J. (2007). Damage Tolerance of Bonded Composite Aircraft Repairs for Metallic Structures. Doctoral Dissertation.

[6] Wen T., Narita F., Kurita H., Jia Y., Shi Y. (2023). Quantification of damage expansion influence on frequency response function of plate for structural health monitoring with integral differential method. Compos. Sci. Technol..

[7] Azad M.M., Kim H.S. (2024). Hybrid deep convolutional networks for the autonomous damage diagnosis of laminated composite structures. Compos. Struct..

[8] Xiao S., Huang M., Xu Z., Yang Y., Du S. (2024). Experimental and Numerical Analysis of Bolted Repair for Composite Laminates with Delamination Damage. Polymers.

[9] Shabani P., Li L., Laliberte J., Qi G. (2024). Compression after impact (CAI) failure mechanisms and damage evolution in large composite laminates: High-fidelity simulation and experimental study. Compos. Struct..

[10] Sonat E., Özerinç S. (2021). Failure behavior of scarf-bonded woven fabric CFRP laminates. Compos. Struct..

[11] Caliskan M. (2006). Evaluation of bonded and bolted repair techniques with finite element method. Mater. Des..

[12] Zhou W., Ji X.-L., Yang S., Liu J., Ma L.-H. (2021). Review on the performance improvements and non-destructive testing of patches repaired composites. Compos. Struct..

[13] Chen S. (2001). Composite Structure Repair Manual.

[14] Hu J.S., Mi S.Q., Yang Z.Y., Wang C.R., Yang Y.H., Tian W. (2022). An experimental investigation on bearing behavior and failure mechanism of bolted composite interference-fit joints under thermal effects. Eng. Fail. Anal..

[15] Ali M., Israr A., Ahmed A., Ikram R. (2023). Effect of Patch Repair on the Physical and Mechanical Properties of Carbon Bidirectional Reinforced Composites. Iran J. Sci. Technol. Trans. Mech. Eng..

[16] Caliskan U., Ekici R., Bayazit A.Y., Apalak M.K. (2021). Numerical model for composite patch repair of notched aluminum plates under impact loading. Proc. Inst. Mech. Eng. Pt. L J. Mater. Design Appl..

[17] Brighenti R., Carpinteri A., Vantadori S. (2006). A genetic algorithm applied to optimisation of patch repairs for cracked plates. Comput. Methods Appl. Mech. Eng..

[18] Karaduman B.N., Elaldi F. (2023). Effects of repair techniques and scarf angles on mechanical performance of composite materials. J. Reinf. Plast. Compos..

[19] Kumar S., Sridhar I., Sivashanker S., Osiyemi S., Bag A. (2006). Tensile failure of adhesively bonded CFRP composite scarf joints. Mater. Sci. Eng. B.

[20] Ghafafian C., Popiela B., Trappe V. (2021). Failure mechanisms of GFRP scarf joints under tensile load. Materials.

[21] Camanho P.P., Davila C.G., De Moura M.F. (2003). Numerical simulation of mixed-mode progressive delamination in composite materials. J. Compos. Mater..

[22] Psarras S., Loutas T., Galanopoulos G., Karamadoukis G., Sotiriadis G., Kostopoulos V. (2020). Evaluating experimentally and numerically different scarf-repair methodologies of composite structures. Int. J. Adhes. Adhes..

[23] Breitzman T., Iarve E., Cook B., Schoeppner G., Lipton R. (2009). Optimization of a composite scarf repair patch under tensile loading. Compos. Part A Appl. Sci. Manuf..

[24] Salve A.K., Jalwadi S.N. Implementation of cohesive zone in ABAQUS to investigate fracture problems. Proceedings of the National Conference for Engineering Postgraduates RIT NConPG–15.

[25] Zhang R., Sun W., Luo H., Ma H., Zhang H. (2024). Finite element dynamic modeling and vibration reduction analysis of the double-lap bolted thin plate with partially attached constrained layer damping. Thin-Walled Struct..

[26] Hashin Z. (1980). Failure Criteria for Unidirectional Fiber Composites. J. Appl. Mech..

[27] Camanho P.P., Dávila C.G., Pinho S.T., Remmers J.J., Rolfes R., Ernst G., Vogler M., Hühne C. (2008). Material and failure models for textile composites. Mechanical Response of Composites.

[28] (2003). Standard Test Method for Measuring the Damage Resistance of a Fiber-Reinforced Polymer Matrix Composite to a Drop-Weight Impact Event.

[29] (2016). Standard Test Method for Compressive Properties of Polymer Matrix Composite Materials with Unsupported Gage Section by Shear Loading.

